# Endovascular aortic repair with sac embolization for the prevention of type II endoleaks (the EVAR-SE study): study protocol for a randomized controlled multicentre study in Germany

**DOI:** 10.1186/s13063-023-07888-8

**Published:** 2024-01-02

**Authors:** Christoph Knappich, Felix Kirchhoff, Marie-Kristin Fritsche, Silvia Egert-Schwender, Heiko Wendorff, Michael Kallmayer, Bernhard Haller, Alexander Hyhlik-Duerr, Christian Reeps, Hans-Henning Eckstein, Matthias Trenner

**Affiliations:** 1 Department for Vascular and Endovascular Surgery, Klinikum rechts der Isar, Technical University of Munich, Munich, 81675 Germany; 2 Münchner Studienzentrum, Klinikum rechts der Isar, Technical University of Munich, Munich, Germany; 3 Klinikum rechts der Isar, Institute of AI and Informatics in Medicine, Technical University of Munich, Munich, Germany; 4 Vascular Surgery, Medical Faculty, University of Augsburg, Augsburg, Germany; 5 Division of Vascular and Endovascular Surgery, Department for Visceral, Thoracic and Vascular Surgery, Medical Faculty Carl Gustav Carus and University Hospital, Technische Universität Dresden, Dresden, Germany; 6 Division of Vascular Medicine, St. Josefs Hospital, Wiesbaden, Germany

**Keywords:** Aortic aneurysm, Endoleak, Endovascular, Embolization, Prevention, Prospective studies

## Abstract

**Background:**

Beyond a certain threshold diameter, abdominal aortic aneurysms (AAA) are to be treated by open surgical or endovascular aortic aneurysm repair (EVAR). In a quarter of patients who undergo EVAR, inversion of blood flow in the inferior mesenteric artery or lumbar arteries may lead to type II endoleak (T2EL), which is associated with complications (e.g. AAA growth, secondary type I endoleak, rupture). As secondary interventions to treat T2EL often fail and may be highly invasive, prevention of T2EL is desirable. The present study aims to assess the efficacy of sac embolization (SE) with metal coils during EVAR to prevent T2EL in patients at high risk.

**Methods:**

Over a 24-month recruitment period, a total of 100 patients undergoing EVAR in four vascular centres (i.e. Klinikum rechts der Isar of the Technical University of Munich, University Hospital Augsburg, University Hospital Dresden, St. Joseph’s Hospital Wiesbaden) are to be included in the present study. Patients at high risk for T2EL (i.e. ≥ 5 efferent vessels covered by endograft or aneurysmal thrombus volume <40%) are randomized to one group receiving standard EVAR and another group receiving EVAR with SE. Follow-up assessments postoperatively, after 30 days, and 6 months involve contrast-enhanced ultrasound scans (CEUS) and after 12 months an additional computed tomography angiography (CTA) scan. The presence of T2EL detected by CEUS or CTA after 12 months is the primary endpoint. Secondary endpoints comprise quality of life (quantified by the SF-36 questionnaire), reintervention rate, occurrence of type I/III endoleak, aortic rupture, death, alteration of aneurysm volume, or diameter. Standardized evaluation of CTA scans happens through a core lab. The study will be terminated after the final follow-up visit of the ultimate patient.

**Discussion:**

Although preexisting studies repeatedly indicated a beneficial effect of SE on T2EL rates after EVAR, patient relevant outcomes have not been assessed until now. The present study is the first randomized controlled multicentre study to assess the impact of SE on quality of life. Further unique features include employment of easily assessable high-risk criteria, a contemporary follow-up protocol, and approval to use any commercially available coil material. Overcoming limitations of previous studies might help SE to be implemented in daily practice and to enhance patient safety.

**Trial registration:**

ClinicalTrials.gov NCT05665101. Registered on 23 December 2022.

## Introduction

### Background and rationale {6a}

Abdominal aortic aneurysm (AAA) is defined as a pathological widening of the aortic diameter to ≥ 3 cm. Over 80% of AAAs are asymptomatic. The most severe complication of AAA is aortic rupture and death with the rupture risk increasing with a rising aortic diameter. AAA prevalence increases with age and ranges between 2–3 and 0.5% in elderly men and women [1, 2]. Since screening is not available for all patients, the true prevalence is expected to be higher [3]. The only preventive strategy for AAA rupture is to perform elective exclusion of the aneurysm once the aortic diameter has reached a critical diameter of 5.5 cm in men and 5.0 cm in women [4, 5].

The hospital incidence of treated AAA in Germany in 2016 was 25.5 and 3.2 per 100,000 male and female residents, respectively. A total of 90% of all AAAs are treated electively, while 10% are treated for ruptured AAA (rAAA). Mortality of rAAA is 80–90% if untreated and 30–50% if treated emergently [5]. Elective AAA repair is associated with mortality rates of 4–6% after open aortic repair (OAR) and 1% after endovascular aortic repair (EVAR) [5].

Open aortic repair (OAR) is done by transabdominal or retroperitoneal approach and by interposition of prosthetic aortic grafts. EVAR is performed by a transfemoral implantation of a stent graft in patients with suitable AAA anatomy. Randomized studies gave evidence that the lower invasiveness of EVAR is associated with lower rates of clinical complications and lower early mortality rates [6]. During longer term follow-up (FU), however, the survival benefit of EVAR decreases, and the survival curves align [7, 8]. This is due to EVAR-associated endoleak (EL), defined as persistent perfusion of the aneurysm sac. Without proper FU and subsequent reinterventions (proximal or distal re-EVAR, coiling of aortic branches, or even conversion to OAR), up to 5% of all AAAs will rupture within 5 years despite previous EVAR [5].

An ongoing clinically worrying problem is the persistence or new occurrence of retrograde perfusion of the AAA sac (T2EL) by lumbar arteries and/or the inferior mesenteric artery (IMA), which occurs in 20–30%. Historically, T2ELs were considered as a clinically irrelevant residual “low-flow” AAA perfusion. Mid- and long-term FU data however demonstrated that about 25% of all T2ELs are associated with secondary complications (e.g. expansion of AAA, shortening of proximal or distal attachment zones with transition to high flow leaks, or even aortic rupture). Therefore, current guidelines recommend endovascular or open treatment of T2ELs if the AAA growth rate exceeds 10 mm/year [5]. However, secondary endovascular treatment of T2EL is often unsuccessful and associated with repeated interventions. Thus, the benefit for patients is unclear [9]. Open ligation of feeder vessels is effective in treatment of T2EL but remains a highly invasive procedure [9, 10].

Lifelong surveillance imaging [i.e. computed tomography angiography (CTA) or contrast-enhanced ultrasound (CEUS)] after EVAR is strongly recommended, but very frequent scans are necessary to detect significant changes in AAA morphology. This leaves patients and physicians alike with a certain degree of uncertainty, which in patients is accompanied by a high mental burden.

In Germany in 2016, around 80% of non-ruptured AAAs were treated by EVAR (8442 cases). Since T2EL occurs in 20–30% and is strongly associated with further complications in 25% of these patients, about 500–700 patients are expected to be affected by T2EL complications each year [11–13].

### Previous trials

Different strategies to prevent T2EL have been proposed in the literature: endovascular aneurysm sealing (EVAS), sac embolization (SE), and side branch embolization (SBE). EVAS involves placement of polymer endo bags in the aneurysm sac. However, due to high failure rates, the device has been voluntarily recalled by the providing company [14–16]. SE and SBE have been evaluated in a meta-analysis, showing promising results (in terms of T2EL prevention and lower re-intervention rates) for both techniques [17]. The main drawback of SBE, however, is that it requires longer radiation times, is more time-consuming, and therefore is frequently performed as a separate procedure [17]. Therefore, it seems unlikely that SBE will be able to achieve wide acceptance among patients and surgeons.

Seven studies on SE have been identified and included in the above-mentioned meta-analysis [18–24], of which only three focussed on patients at high risk for developing T2EL, using diverging high-risk criteria [18, 19, 22]. One of these studies randomized patients for SE (volume-dependent use of coils and fibrin glue) vs. non-SE [22]. The very widely defined high-risk criteria led to the inclusion of almost all EVAR patients (85%). After 3 months, SE showed lower T2EL rates (20% vs. 41%); however, due to a high number of reinterventions in the non-SE group, the 24-month T2EL rates were very similar (13% vs. 16%) [22].

More recently, another randomized controlled trial (RCT) was published [25], defining high-risk criteria as a patent IMA with a diameter of > 3 mm, at least three pairs of patent lumbar arteries, or two pairs of lumbar arteries and a median sacral artery or an accessory renal artery. Different from our approach, the thrombus was not evaluated as high-risk factor, and the material used for coiling was limited to only one specific manufacturer and type of coil. Follow-up was conducted by plain duplex ultrasound and CTA until 24 months. Ninety-four patients were randomized. T2EL rates were lower in the SE group for up to 12 months (41% vs. 14%), whereas the difference lost statistical significance at 24 months (25% vs. 6.5%). Survival free from EL and re-interventions was significantly in favour of the SE group (*p* < 0.001), and aneurysm sac volume decreased significantly in the respective group at 6, 12, and 24 months.

The 2019 guidelines on AAA management by the *European Society for Vascular and Endovascular Surgery (ESVS)* encouraged further investigation in the field of SE: “Pre-operative sac embolization in selected patients has been suggested as a technique to reduce risk of T2EL development during follow up, but the benefit of a reduced number of late re-interventions or decreased incidence of rupture remains to be proven” [5].

Risk factors for development of T2EL have been described in a meta-analysis on 15 prospective and retrospective studies. The overall prevalence of T2EL was 22% [12]. Probably due to better FU imaging, the reported prevalence increased in studies during the last decade to even 27%. The following clinical and anatomical variables were significantly associated with T2EL: age (younger vs. older: *OR* 0.37), smoking (vs. non-smoking: *OR* 0.71), patency of an IMA (*OR* 1.98), number of covered patent lumbar arteries (*OR* 3.07), and maximal AAA diameter (smaller vs. larger: *OR* 0.23) [12]. Moreover, regarding the persistence of T2EL and sac expansion, the number of patent lumbar arteries (≥ 4–6), patent IMA, and intraluminal thrombus volume (< 40%) is the most important prognostic factors [26, 27].

To define inclusion criteria for the planned study, the above-mentioned criteria were evaluated in a retrospective analysis of 100 consecutive patients treated at Klinikum rechts der Isar in 2016–2018. The presence of ≥ 5 patent efferent vessels and/or < 40% thrombus at largest AAA diameter (see formula in inclusion criteria) showed a 100% sensitivity in predicting T2EL, with the specificity being 45%. Other combinations of potential predictors for T2EL demonstrated higher specificities but lower sensitivities.

### Objectives {7}

The primary aim of the EVAR-SE study is to assess whether AAA SE using metal coils is effective in patients at high risk for developing T2EL after EVAR.

Secondary objectives are to assess the safety of AAA SE and effects on quality of life, AAA growth, occurrence of type I/III endoleaks, and reintervention rates.

### Trial design {8}

**Fig. 1:**
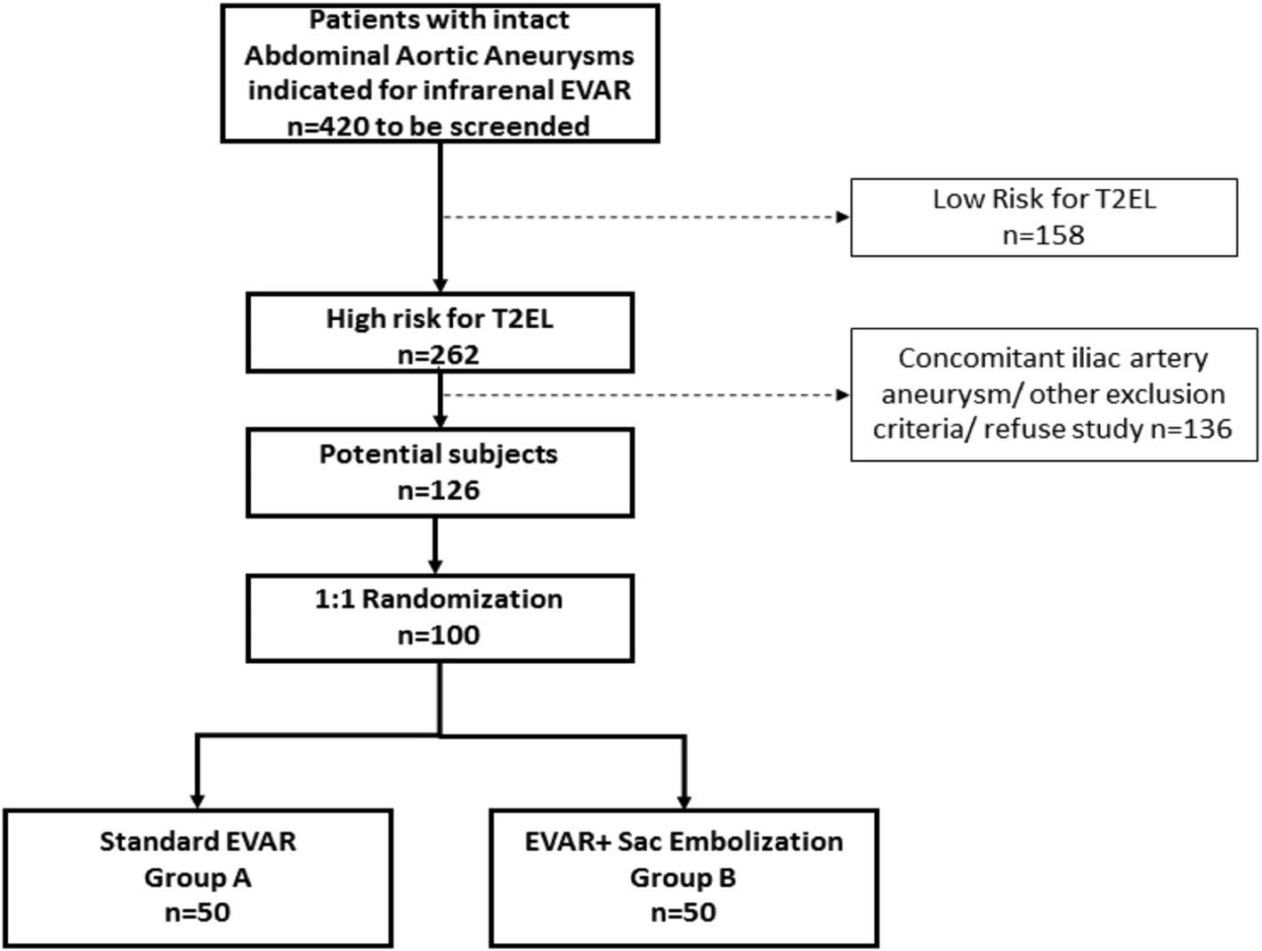
Estimated patient flow chart illustrating patient populations provisionally to be screened, to be excluded, to be included, and to be randomized within the EVAR-SE study. EVAR, endovascular aortic repair; T2EL, type 2 endoleak

This study protocol describes an exploratory prospective, 2-armed, randomized, parallel-group multicentric clinical study to evaluate superiority of AAA SE with coils in combination with standard EVAR over standard EVAR without SE (Fig. 1).

## Methods: participants, interventions, and outcomes

### Study setting {9}

The study is conducted at four dedicated vascular surgery centres in Germany. Besides, the Klinikum rechts der Isar of the Technical University of Munich, the University Hospitals of Augsburg and Dresden, and St. Josef’s Hospital Wiesbaden participate as recruiting sites. The study is conducted in conjunction with the Münchner Studienzentrum (MSZ; Clinical Trials Center at School of Medicine, Technical University of Munich).

### Eligibility criteria {10}

To be eligible for study inclusion, patients to be included must have reached 18 years of age and must be diagnosed with a AAA measuring ≥ 50 mm and justifying an indication for EVAR within the instructions for use (IFU) proposed by the manufacturer. They may only be included if fulfilling one or both high-risk criteria for T2EL on CTA. Those are defined as either ≥ 5 patent efferent vessels (e.g. IMA, lumbar arteries, median sacral artery, accessory renal arteries) provisionally covered by the stent graft or the amount of thrombus at the largest AAA diameter being < 40% according to the auxiliary formula:

$$thromus\ \left(\%\right)=1-\frac{\textrm{patent}\ \textrm{aortic}\ \textrm{lumen}\ \textrm{diameter}}{\textrm{maximal}\ \textrm{aortic}\ \textrm{diameter}}$$

Patients must not be included if suffering a rAAA or concomitant iliac artery aneurysm disease, if their AAA requires fenestrated or branched AAA, if unable to adhere to the FU protocol, or in the cases of pregnancy or lack of consent.

### Who will take informed consent? {26a}

Informed consent will be obtained from staff physicians who concurrently are specifically trained and accredited members of the study team.

### Additional consent provisions for collection and use of participant data and biological specimens {26b}

The informed consent form contains a section on data usage and processing which is to be signed separately by each participant. No biological specimens will be collected.

## Interventions

### Explanation for the choice of comparators {6b}

EVAR with SE is to be compared to standard EVAR, as the latter is widely accepted as the standard of care.

### Intervention description {11a}

**Fig. 2:**
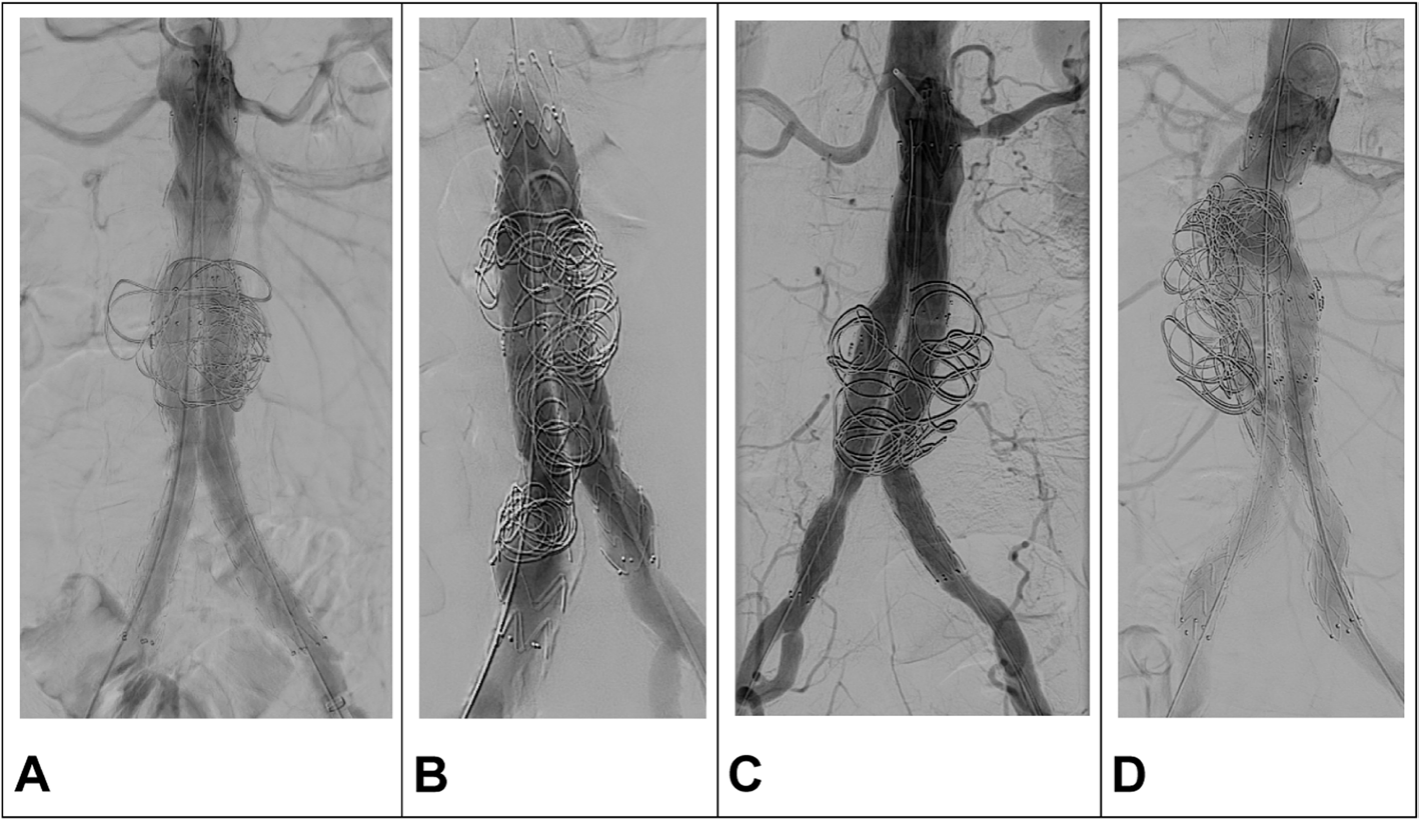
Examples of intraoperative angiograms after endovascular aortic repair with sac embolization. Within the EVAR-SE study, a minimum of 2 m of coil material is to be implanted in the abdominal aortic aneurysm sac. It is aimed for homogeneous filling of the aneurysm sac with coils forming a lose network to trigger thrombus formation

SE in the intervention group is conducted during the standard EVAR procedure. Access to the aneurysm sac is gained by an angiographic catheter placed in the sac before deployment of the EVAR stent graft, either by using an accordingly oversized sheath for implantation of the contralateral limb graft or by an additional puncture and insertion of a 4F sheath. After full deployment of the aortic stent graft and bilateral limb grafts, a minimum of 2 m of coils are implanted in the AAA sac (Fig. 2). If the impression of the operating surgeon is that more coil material is needed for filling the sac, additional coils will be implanted. Thereafter, the angiography catheter is retracted from the AAA sac, and all attachment and connection zones are balloon dilated to warrant sufficient sealing.

Involved investigators have received a training course for the SE procedure and are asked to follow a clear standard operating procedure (SOP). In doing so, the choice of stent graft and coils will be left to the discretion of the operating surgeon. The procedure is simply applicable and easy to adopt for certified vascular surgeons; thus, the expected learning curve is expected to be negligible but will be statistically evaluated.

### Criteria for discontinuing or modifying allocated interventions {11b}

Not applicable, as allocated interventions will provisionally be executed without exceptions.

### Strategies to improve adherence to interventions {11c}

Not applicable, as allocated interventions will provisionally be executed without exceptions.

### Relevant concomitant care permitted or prohibited during the trial {11d}

Not applicable, as no relevant concomitant care is permitted during the trial.

### Provisions for posttrial care {30}

Not applicable, as suffering harm from trial participation is highly unlikely.

### Outcomes {12}

#### Primary endpoint

Evaluation of the T2EL proportion of the intervention group as compared to the control group at 12 months after EVAR treatment as measured by CEUS and/or CTA.

The primary endpoint was chosen, since under the presence of a T2EL, there is a risk of developing further complications in the longer term. Ultimately, in a confirmatory study, SE will have to be tested in a setting with longer-term follow-up (5 or even 10 years) and mortality as primary endpoint. However, in the setting of this exploratory study, SE is not expected to relevantly influence mortality for the treated patients within 12 months of follow-up. Mortality will be evaluated as a secondary endpoint in this trial.

#### Secondary endpoints

The following secondary endpoints will be investigated and compared between study groups:

- T2EL proportions at time points 2–4 days, 30 days and 6 months after EVAR treatment, as measured by CEUS and/or CT-A
- Rates of any re-intervention (for endoleaks, occlusions, graft infection, graft migration)
- Occurrence of any EL types I or III
- Changes of AAA diameter and volume as measured by CTA in comparison to the initial CTA scan at V0
- Rate of AAA rupture 12 months after EVAR treatment
- Mortality (aneurysm related/not aneurysm related) 12 months after EVAR treatment
- Change in quality of life assessed by SF-36 from baseline to 12 months after EVAR treatment

### Participant timeline {13}

The frequency of study visits is equal to standard follow-up protocols after EVAR implantation (Table 1). CEUS will be used to determine the presence of an EL at 2–4 days, 30 days, 6 months, and 12 months after surgery. At 12 months, a CTA scan will be used as further imaging method. Patients additionally will be asked to answer a quality-of-life questionnaire after 12 months using a standard evaluation form (SF-36) [28].

**Table 1:** Schedule of parameters and study visits. Visit timelines follow clinical routine

Visit	Screening/baseline	V0	V1a	V1b	V2	V3	V4
Months (m)/days (d)	−1–0 m		0–2 months (EVAR)	2–4 days after V1a	30 days	6 months	12 months
Eligibility criteria	+						
Informed consent ^a^		+					
Medical history ^b^, baseline characteristics ^c^		+					
Concomitant medication (esp. anticoagulation) ^d^		+	+^h^	+^h^	+^h^	+^h^	+
Randomization		+					
EVAR procedural details ^e^			+				
Details on AAA morphology ^f^/volume (CT-A), the presence of EL		+					+
The presence of EL, AAA diameter (CEUS)				+	+	+	+
Renal function ^g^		+		+			+
Adverse events			+	+	+	+	+
Reintervention				+	+	+	+
Quality of life (SF-36)		+					+

*CT-A* computer tomography with angiography, *CEUS* contrast-enhanced ultrasound. ^a ^The presence of a valid informed consent must be checked at the indicated study visits. ^b^ Medical history includes information on comorbidities (e.g. coronary heart disease, cerebrovascular disease, peripheral arterial disease, chronic obstructive pulmonary disease, malignancies, diabetes, arterial hypertension) and smoking habit. ^c^ Baseline characteristics include patient’s age and weight. ^d^ Medication includes information on antihypertensives, lipid-lowering agents, antiplatelet medication, and anticoagulation. ^e^ Procedural details include operation time and fluoroscopy time; in arm B, information on coils (e.g., type, length, number) and EVAR prosthesis (e.g. type, diameter). ^f^ Details on AAA morphology include length of neck, AAA diameter, number of efferent vessels (e.g. lumbar arteries, accessory renal arteries). ^g^ Renal function parameters include serum creatinine, serum urea, glomerular filtration rate. ^h^On these visits, only data on anticoagulation and antiplatelet medication will be captured

### Sample size {14}

The primary aim is to reduce the proportion of T2EL in high-risk patients 12 months after the index procedure. The T2EL proportion in the control group after 1 year is assumed to be 45%. This assumption is based on a retrospective analysis of 100 consecutive patients treated at Klinikum rechts der Isar. The expected proportion of T2EL in the intervention group is 15%. This translates to an odds ratio of 0.22. The expected T2EL rates after 12 months in the respective groups are in accordance with the above mentioned RCT (41% vs. 14%; adjusted *OR* 0.19; 95% *CI* 0.05–0.71) [25].

Under these assumptions, 42 patients per group (84 overall) will be needed to detect a difference between the treatment groups with a statistical power of 80% (two-sided continuity-corrected chi-squared test, *α* = 5%). Due to potential loss to FU or incomplete FU, a total number of 50 patients per group is planned to be allocated to the study. Sample size calculation was performed using the software nQuery Advisor 7.0.

### Recruitment {15}

Any patient who is to undergo EVAR for infrarenal AAA will be evaluated for eligibility. In- and exclusion criteria are detailed above. In order to document the proportion of patients that were included from patients screened, a screening list is kept and updated in each centre.

## Assignment of interventions: allocation and blinding

### Sequence generation {16a}

The randomization sequence was created by MSZ using RANCODE professional 2015. Randomization is performed block wise using varying block sizes and stratified by centre.

### Concealment mechanism {16b}

A box with envelopes containing the respective treatment arm is available at the respective study centre.

### Implementation {16c}

The randomization sequence was created by MSZ. Participants will be enrolled and allocated to interventions by the responsible study nurse of the respective study centre.

### Blinding {17a}

Operator blinding is not possible, the same accounts for blinding of FU assessments, as coils are visible on imaging modalities (i.e. CEUS and CTA). Participants will not be blinded, as this would impede the assessment of SE on quality of life.

### Procedure for unblinding if needed {17b}

Not applicable, as no blinding will be executed.

## Data collection and management

### Plans for assessment and collection of outcomes {18a}

The documentation of the study data is the responsibility of the local investigators. Original data (source documents) remain at the respective study site. Medical record and information on the eCRF must be traceable and consistent with the original data. All data collected in this study must be entered in the eCRF which has to be completed by the investigator or authorized study personnel and signed by the investigator. The site investigators are responsible for ensuring the accuracy, completeness, and timeliness of all data reported to the study leadership in the eCRFs and in all required reports.

### Plans to promote participant retention and complete follow-up {18b}

A standardized section will be included in every participant’s medical letter to inform his/her general physician on his/her patient’s participation in the study and on the importance to strictly adhere to all follow-up visits.

### Data management {19}

After database lock, the investigators will receive the data of their respective study centre. Data are administered and processed by data management of the MSZ with the support of a study database (eCRF). The evaluation of the data takes place by programmed validity and consistency checks. In addition, a manual/visual evaluation of plausibility is performed. After entry of all collected data in this study and clarification of all queries, the database will be closed. Data and results electronically recorded will be archived according to applicable legal requirements.

### Confidentiality {27}

The applicable regulations of data privacy protection will be followed. The confidentiality of records that could identify subjects will be protected, respecting the privacy and confidentiality rules in accordance with the applicable regulatory requirement(s). The patients will be informed that any patient-related data and materials will be appropriately made pseudonymous, and that these data may be used for analysis and publication purposes. Furthermore, the patients will be informed that their data may be inspected by monitors or other authorized personnel. Patients who do not provide consent for transmission of their data, according to the data protection agreement included in the ICF, will not be included in the study.

### Plans for collection, laboratory evaluation, and storage of biological specimens for genetic or molecular analysis in this trial/future use {33}

Not applicable, as no biological specimens will be collected.

## Statistical methods

### Statistical methods for primary and secondary outcomes {20a}

Primary analysis will be performed following the intention-to-treat (ITT) principle, so each participant will be analyzed in the group he or she was randomized to. Absolute and relative frequencies will be determined for the primary endpoint (T2EL 12 months after index procedure) for both study groups. For patients that do not have a valid T2EL assessment at Visit 4 (12 months, including patients who died unrelated to the index procedure), the result of the 6-month evaluation will be imputed. A multiple imputation approach will be performed to impute missing values of participants with missing assessments at Visit 3 and Visit 4 (6 months and 12 months after index procedure, including patients who died unrelated to the index procedure before Visit 3). Age, sex, and study group will be considered in the imputation approach. Logistic regression models with the presence of T2EL at 12 months as dependent variable and study group, age, and sex as independent variables will be fitted to the imputed datasets. Results will be aggregated following Rubin’s rules, and an odds ratio with corresponding 95% confidence interval will be estimated and presented. Due to the small number of expected events, study centre will not be included as independent variable in the model. Sensitivity analyses based on complete cases only and on a per protocol set including only patients that were treated as described in the surgery SOP for their allocated study group will be conducted. Additionally, a time-to-event analysis considering death as competing event will be performed as sensitivity analysis.

Distributions of time to re-intervention, time to occurrence of endoleaks I/III, time to AAA rupture, and time to death will be estimated by Kaplan-Meier method or cumulative incidence function if competing risks are present. Logrank tests will be performed for group comparisons of (cause-specific) hazard rates. Categorical outcomes will be compared using Fisher’s exact tests or continuity-corrected chi-squared tests. For changes in quantitative outcomes (AAA diameter, quality of life), linear regression models with the corresponding change as dependent variable and study group and the baseline value as independent variables will be fitted to the data. All secondary endpoints will be analyzed in an exploratory manner. Effect measures (hazard ratios, odds ratios, mean differences) will be presented with corresponding 95% confidence intervals.

### Interim analyses {21b}

No interim analysis will be performed, unless demanded by the safety monitoring board (SMB).

### Methods for additional analyses {20b}

A subgroup analysis comparing treatment effects of the early study phase (patients recruited in the first year) with the late study phase (second year) will be performed. A logistic regression model including study group, study phase, and their interaction term will be fitted to the data to test for treatment effect heterogeneity in order to assess the presence of a learning effect. In an additional subgroup analysis, results will be shown stratified for study centre using the same approach as described for study phase.

### Methods in analysis to handle protocol non-adherence and any statistical method to handle missing data {20c}

For patients that do not have a valid T2EL assessment at Visit 4 (12 months, including patients who died unrelated to the index procedure), the result of the 6-month evaluation will be imputed. A multiple imputation approach will be performed to impute missing values of participants with missing assessments at Visit 3 and Visit 4 (6 months and 12 months after index procedure, including patients who died unrelated to the index procedure before Visit 3). Age, sex, and study group will be considered in the imputation approach.

### Plans to give access to the full protocol, participant level-data, and statistical code {31}

The full protocol, datasets collected during the current study, and the statistical code will be available from the corresponding author on reasonable request.

## Oversight and monitoring

### Composition of the coordinating centre and trial steering committee {5d}

The trial steering committee consists of the study leader, the statistician, and two senior physicians who have been strongly involved in conception and execution of the study. While the steering committee is meeting approximately every 4 weeks, the team running the trial day to day (one of both above-mentioned two senior physicians together with one junior physician and a study nurse) are meeting every other day and always if required.

### Composition of the data monitoring committee and its role and reporting structure {21a}

Monitoring activities are performed by monitors of the MSZ to ensure that the study is conducted in accordance with the protocol and ethical requirements. A monitoring plan describing the scope of the monitoring activities in detail will be prepared.

The monitor will have access to patient records, any information needed to verify the entries in the eCRF, and all necessary information and essential study documents. The investigator will cooperate with the monitor to ensure that any problems detected in the course of these monitoring visits are resolved. A monitoring visit report will be prepared for each visit describing the progress of the study and all identified problems.

### Adverse event reporting and harms {22}

All adverse events and severe adverse events (SAE) must be documented in the eCRF. SAEs have to be documented within 24 h. Adverse events and SAEs (including information on severity, seriousness, start and stop date) are reported immediately to the responsible monitor of the study centre by an automatically generated email.

### Risks

SE in the intervention group will be conducted during the standard EVAR procedure according to standard techniques.

The procedure itself requires insertion of a slightly oversized sheath or puncture with an additional 4F sheath. Depending on the device, standard EVAR usually requires a 14–16F access to implant the contralateral limb. Access site closure is routinely achieved using arterial closure devices certified for this procedure. The device used should be approved for closure of large bore arterial access up to 21F (26F outer diameter). A retrospective analysis of patients in the Italian Percutaneous EVAR (IPER) registry did not show a significant association between > 18F access and technical failure of percutaneous access [29]. Equally, a retrospective study on 266 patients undergoing percutaneous EVAR using the ProGlide™ system did not show an association between sheath size and groin complications [30]. In conclusion, the totality of the available evidence suggests the sheath size seems not to be associated with a significant elevation of access site complications. Therefore, we estimate the additional access-related risk for patients undergoing SE in the EVAR-SE study to be negligible.

In our experience, there have been rare cases of coil displacement into the iliac artery. In all cases, it was possible to remove the coil by endovascular means not leaving foreign material in the blood stream.

The SE procedure was shown to be associated with a 7–18 min increased operating time [17]. As the whole operation is performed percutaneously without open surgical access, the additional operating time is not expected to be associated with a higher rate of surgical site infections.

Furthermore, SE is accompanied by an additional 2–7 min of fluoroscopy time [17]. However, it was shown that the body irradiation dose was not significantly increased compared to EVAR alone [25]. The SE procedure usually is not associated with additional use of contrast media.

### Frequency and plans for auditing trial conduct {23}

An independent SMB was established. The underlying principles for the SMB are ethical and safety aspects. The SMB examines whether the conduct of the study is still ethically justifiable and whether safety of the patients is ensured. For this, the SMB is informed regularly about patient recruitment and observed safety events. Serious adverse events (SAE) are recorded on a study-specific safety form within the study database. SAE documentation is processed in written form to the SMB and the ethics committees.

### Termination criteria

Individual patient criteria resulting in termination of the study involve indications for explantation of the endograft (e.g. endoleak type I, graft infection), adverse events that make adherence to follow up unacceptable, severe kidney injury or allergic reactions to contrast media, and withdrawal of consent.

The study may be terminated in the respective study centre in case of insufficient patient recruitment, major protocol violations, or noncompliance with study requirements.

In the event of safety concerns, failure of a relevant number of recruitment centres to comply with the protocol, or inadequate recruitment of subjects, the whole trial may be terminated.

### Withdrawals

Patients who wish to withdraw from the study may do so at any point. In this case, no further data will be collected, while already collected data might need to be discarded upon the patient’s wish. Withdrawn participants will be replaced in order to reach the projected sample size.

### Registration

The EVAR-SE study was registered on the ClinicalTrials.gov public website on 23 December 2022. The ClinicalTrials.gov identifier is NCT05665101.

### Plans for communicating important protocol amendments to relevant parties (e.g. trial participants, ethics committees) {25}

All relevant protocol amendments will be communicated to the ethics committees of the participating centres, ClinicalTrials.gov, and the journal in which the study protocol was publishes.

### Dissemination plans {31a}

The study protocol hereby is published in a peer-reviewed scientific journal. Study results will be prepared for publication in a reputable peer-reviewed scientific journal in accordance with the CONSORT statement [31]. Further, results will be disseminated in lay language to patients and public society. The BMBF will be acknowledged as funder in all publications and presentations by providing the BMBF project number 01KG2128.

### Good clinical practice

The study is carried out in accordance with the principles of the Declaration of Helsinki by the World Medical Association and specific applicable national ethical and regulatory requirements [“Berufsordnung für Ärzte”, General Data Protection Regulation (GDPR)] [32]. Moreover, an independent SMB is established.

## Discussion

The short-term outcome of standard EVAR, with in-hospital mortality rates of less than 1% and low rates of primary complications, makes EVAR the first-choice treatment for patients and surgeons alike. The long-term success of the treatment is, however, endangered by T2EL, which occur in one out of four patients. By identifying patients at higher risk of T2EL and taking sac embolization as preventive measure, the incidence of T2EL including their potential complications might be reduced.

Sac embolization has shown encouraging results in other studies [17], especially the recently published randomized SCOPE trial [25]. This might lead to the question why a further exploratory study is necessary before conducting a confirmatory study with long-term follow up (5–10 years) and aneurysm-related survival as primary endpoint. All previous studies hold major drawbacks that we are planning to overcome with EVAR-SE:

### Primary endpoint

The conductors of SCOPE admit as major limitation that the primary endpoint should have been uniform for all patients, rather than at different timelines (1, 6, 12, and 24 months) [25]. The primary endpoint for EVAR-SE will be the T2EL proportion of the intervention group as compared to the control group at 12 months after EVAR treatment, as only very few endoleaks occur or seal at a later time point.

### Choice of coils should not be limited to one medical product

Previous RCTs comparing EVAR with SE to standard EVAR allowed only for predefined coil manufacturers [22, 25]. The aim of EVAR-SE is to evaluate the SE method in an easily convertible setting. We therefore plan to allow for CE-certified coils from various industrial manufacturers.

### CEUS as primary follow up imaging

For the SCOPE trial — similar to other earlier studies — patients received at least five CTAs within 2 years (routine planning CTA for EVAR, further scans at 1, 6, 12, and 24 months), combined with color-Doppler ultrasound. For EVAR-SE, a contemporary follow-up protocol will be implemented, replacing most CTA scans by CEUS. Patients will be exposed to less radiation and iodine contrast agent (comparable or less than routine care conditions). CEUS, when compared to CTA, has already been proven to be as sensitive in detecting endoleaks, especially T2EL [33]. It can also detect delayed late T2EL, possibly missed by CTA [34]. CEUS also performs markedly better in detecting endoleaks compared to color-Doppler ultrasound [35]. Thus, CEUS is currently recommended as primary imaging method after EVAR, while CTA should only be performed in the presence of sac enlargement or endoleak [36]. In the special situation, after SE, currently, there is no published data on the use of CEUS as FU imaging. CTA, however, can be hard to interpret, as the implanted coils are causing artefacts [25]. In our own experience, CEUS works well when scanning aneurysm sacs after SE and might help to overcome this issue.

### Adherence to follow-up

Many patients did not attend planned follow-up examinations in SCOPE for unknown reasons [25]. For EVAR-SE, we conducted a patient survey with promising results regarding adherence to follow-up protocols.

### Patient relevant outcome measures

Earlier studies lacked patient relevant outcomes, while EVAR-SE will include quality of life as secondary outcome measure.

### Evaluation in CTA by independent core lab

An independent core lab, consisting of a vascular surgeon and a radiologist (both otherwise not involved in the conduct of the study), will be implemented. The core lab will evaluate pseudonymized CTA scans (before EVAR and after 12 months).

In summary, the method, as applied in EVAR-SE, is technically easy and potentially applicable for everyday use. Thus, if proven efficient, it might find wide acceptance in clinical practice and will be relevant for future AAA treatment guidelines.

## Current trial status

The latest protocol version (number 1.1; 24.11.2022) was approved by the domestic Ethics Committee of the Technical University of Munich in December 2022 and all other ethics committees of the respective study sites (respective reference numbers see below).

Initiation was completed at Klinikum rechts der Isar of the Technical University of Munich in December 2022, at St. Joseph’s Hospital Wiesbaden in June 2023, at the University Hospital Dresden in August 2023, and at the University Hospital Augsburg in September 2023.

The first patient was recruited on 13th January 2023. Until today (1st October 2023), a total of 19 patients have been randomized. Recruitment is estimated to be complete in the 1st quarter of 2025.

## Abbreviations

AAA: Abdominal aortic aneurysm
BMBF: Bundesministerium für Bildung und Forschung
CEUS: Contrast-enhanced ultrasound
CONSORT: Consolidated Standards of Reporting Trials
CT-A: Computed tomography angiography
ESVS: European Society for Vascular and Endovascular Surgery
eCRF: Electronic case report form
EVAR: Endovascular aortic repair
EVAS: Endovascular aortic sealing
FU: Follow-up
GDPR: General Data Protection Regulation
IFU: Intention for use
IMA: Inferior mesenteric artery
IPER: Italian Percutaneous EVAR registry
ITT: Intention to treat
MSZ: Münchner Studienzentrum
OAR: Open aortic repair
QoL: Quality of life
rAAA: Ruptured abdominal aortic aneurysm
SAE: Severe adverse event
SBE: Side branch embolization
SMB: Safety monitoring board
SCOPE: Sac COil embolisation for Prevention of Endoleak
SE: Sac embolization
SF-36: Short-Form 36 Health Survey Questionnaire
SOP: Standard operating procedure
T2EL: Type II endoleak
